# Supplementary material for: Empirical methods for controlling false positives and estimating confidence in ChIP-Seq peaks
Source: BMC Bioinformatics. 2008 Dec 5;9:523. doi: 10.1186/1471-2105-9-523 (PMC2628906; doi:10.1186/1471-2105-9-523)
Supplement: Additional File 1 — A variety of html documents from the USeq web site detailing the available applications, their best usage, output file type descriptions, command line menus, etc. [file 1471-2105-9-523-S1.zip › USeqUserGuides/JavaDoc.zip!/JavaDoc/gata/aligner/AlignerThread.html]

AlignerThread


|  |  |  |
| --- | --- | --- |
| **Overview** \| **Package** \| **Class** \| **Use** \| **Tree** \| **Deprecated** \| **Index** \| **Help** | |  |
| **PREV CLASS**   **NEXT CLASS** | **FRAMES**    **NO FRAMES**     **All Classes** |  |
| SUMMARY: NESTED \| FIELD \| CONSTR \| METHOD | DETAIL: FIELD \| CONSTR \| METHOD |  |


---


## gata.aligner Class AlignerThread

```
java.lang.Object
  java.lang.Thread
      gata.aligner.AlignerThread
```

**All Implemented Interfaces:**: java.lang.Runnable

---

``` public class AlignerThread extends java.lang.Thread ```

**Author:**
:   nix
    Threaded version of primary GATAligner methods to actually perform and save the alignment.
    Updates the progress monitor.

---

**Nested Class Summary**

**Nested classes/interfaces inherited from class java.lang.Thread**

`java.lang.Thread.State, java.lang.Thread.UncaughtExceptionHandler`


**Field Summary**

**Fields inherited from class java.lang.Thread**

`MAX_PRIORITY, MIN_PRIORITY, NORM_PRIORITY`


**Constructor Summary**

`AlignerThread(javax.swing.ProgressMonitor pm,
AlignerInputForm aif)`


| **Method Summary** | |
| --- | --- |
| `void` | `run()` |
| `boolean` | `setProgMon(java.lang.String newNote, int progress, javax.swing.ProgressMonitor pm)` |

**Methods inherited from class java.lang.Thread**

`activeCount, checkAccess, countStackFrames, currentThread, destroy, dumpStack, enumerate, getAllStackTraces, getContextClassLoader, getDefaultUncaughtExceptionHandler, getId, getName, getPriority, getStackTrace, getState, getThreadGroup, getUncaughtExceptionHandler, holdsLock, interrupt, interrupted, isAlive, isDaemon, isInterrupted, join, join, join, resume, setContextClassLoader, setDaemon, setDefaultUncaughtExceptionHandler, setName, setPriority, setUncaughtExceptionHandler, sleep, sleep, start, stop, stop, suspend, toString, yield`

**Methods inherited from class java.lang.Object**

`equals, getClass, hashCode, notify, notifyAll, wait, wait, wait`

**Constructor Detail**

### AlignerThread

```
public AlignerThread(javax.swing.ProgressMonitor pm,
                     AlignerInputForm aif)
```


**Method Detail**

### run

```
public void run()
```

:   **Specified by:**: `run` in interface `java.lang.Runnable` **Overrides:**: `run` in class `java.lang.Thread`

---


### setProgMon

```
public boolean setProgMon(java.lang.String newNote,
                          int progress,
                          javax.swing.ProgressMonitor pm)
```


---


|  |  |  |
| --- | --- | --- |
| **Overview** \| **Package** \| **Class** \| **Use** \| **Tree** \| **Deprecated** \| **Index** \| **Help** | |  |
| **PREV CLASS**   **NEXT CLASS** | **FRAMES**    **NO FRAMES**     **All Classes** |  |
| SUMMARY: NESTED \| FIELD \| CONSTR \| METHOD | DETAIL: FIELD \| CONSTR \| METHOD |  |


---
